# Primaquine–quinoxaline 1,4-di-*N*-oxide hybrids with action on the exo-erythrocytic forms of *Plasmodium* induce their effect by the production of reactive oxygen species

**DOI:** 10.1186/s12936-019-2825-8

**Published:** 2019-06-19

**Authors:** Leonardo Bonilla-Ramírez, Silvia Galiano, Miguel Quiliano, Ignacio Aldana, Adriana Pabón

**Affiliations:** 1 Grupo Malaria, Facultad de Medicina, Universidad de Antioquia (UdeA), Sede de Investigación Universitaria (SIU), Medellín, Colombia; 2 GIEPRONAL, Escuela de Ciencias Básicas Tecnología e Ingeniería, Universidad Nacional Abierta y a Distancia, Medellín, 050012 Colombia; 3 Institute of Tropical Health (ISTUN), Universidad de Navarra, Campus Universitario, 31008 Pamplona, Spain; 4 Department of Organic and Pharmaceutical Chemistry, Universidad de Navarra, Facultad de Farmacia y Nutrición, Campus Universitario, 31008 Pamplona, Spain; 5 Centre for Research and Innovation, Faculty of Health Sciences, Universidad Peruana de Ciencias Aplicadas (UPC), 15023 Lima, Peru

**Keywords:** Malaria, *Plasmodium*, Exoerythrocytic stage, Quinoxaline 1, 4-Di-*N*-oxide, Cell death, Oxidative stress

## Abstract

**Background:**

The challenge in anti-malarial chemotherapy is based on the emergence of resistance to drugs and the search for medicines against all stages of the life cycle of *Plasmodium* spp. as a therapeutic target. Nowadays, many molecules with anti-malarial activity are reported. However, few studies about the cellular and molecular mechanisms to understand their mode of action have been explored. Recently, new primaquine-based hybrids as new molecules with potential multi-acting anti-malarial activity were reported and two hybrids of primaquine linked to quinoxaline 1,4-di-*N*-oxide (PQ–QdNO) were identified as the most active against erythrocytic, exoerythrocytic and sporogonic stages.

**Methods:**

To further understand the anti-malarial mode of action (MA) of these hybrids, hepg2-CD81 were infected with *Plasmodium yoelii* 17XNL and treated with PQ–QdNO hybrids during 48 h. After were evaluated the production of ROS, the mitochondrial depolarization, the total glutathione content, the DNA damage and proteins related to oxidative stress and death cell.

**Results:**

In a preliminary analysis as tissue schizonticidals, these hybrids showed a mode of action dependent on peroxides production, but independent of the activation of transcription factor p53, mitochondrial depolarization and arrest cell cycle.

**Conclusions:**

Primaquine–quinoxaline 1,4-di-*N*-oxide hybrids exert their antiplasmodial activity in the exoerythrocytic phase by generating high levels of oxidative stress which promotes the increase of total glutathione levels, through oxidation stress sensor protein DJ-1. In addition, the role of HIF1a in the mode of action of quinoxaline 1,4-di-*N*-oxide is independent of biological activity.

## Background

Malaria is one of the world’s most important tropical parasitic diseases. Despite strategies for vector control and artemisinin-based combination therapy (ACT), in 2017 an estimated 219 million cases of malaria and 435,000 deaths occurred in worldwide [1]. In the context of malaria elimination, looking for new drugs or effective therapies in all parasites and stages would be a good approach to achieve this goal.

The development of anti-malarial chemotherapy has been focused on the erythrocytic stage of the malaria parasite, from quinine to artemisinin and its derivatives. However, anti-malarial drug resistance has been a recurrent problem and one of the main obstacles in the fight against malaria. In recent years, the emergence of artemisinin resistance has been confirmed [2, 3]. Additionally, the exoerythrocytic or hepatic stage has been poorly characterized, only, primaquine (PQ) and tafenoquine^GSK^, the latter, recently approved by the FDA are unique drugs for the treatment of relapsing malaria and eliminating this exoerythrocytic forms (EEF) in the infections with *Plasmodium vivax* and *Plasmodium ovale* [4, 5].

A strategy to search new compounds with anti-malarial activity is the synthesis of hybrids, which combine more than one pharmacophore in a single molecule [6, 7]. Several hybrids with primaquine moiety with good anti-malarial activity have been reported in the past decades [8–10].

**Fig. 1:**
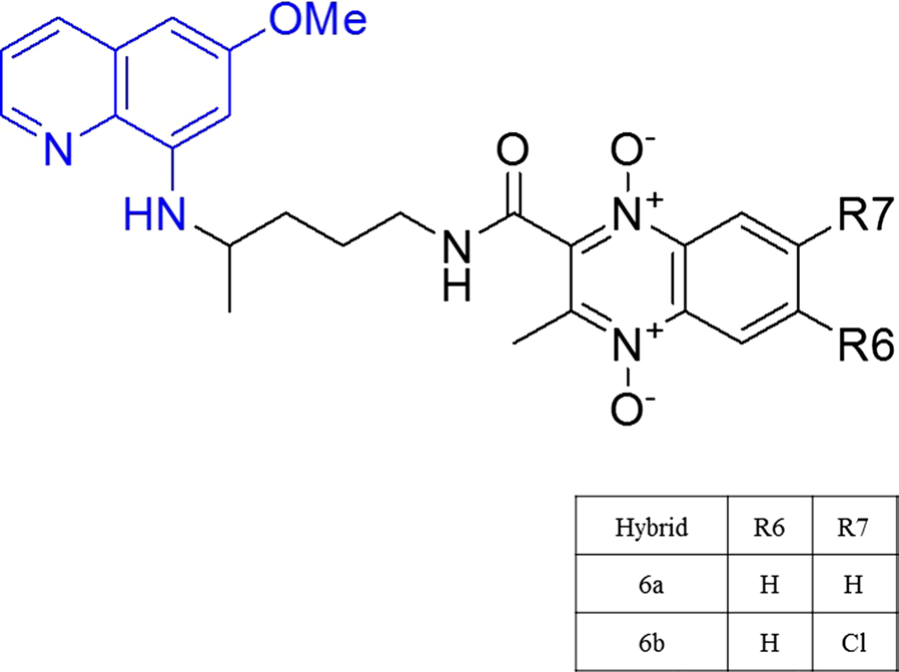
The general structure of PQ–QdNO hybrids. Schematic representation of PQ–QdNO hybrids showed. The moiety primaquine (blue) and quinoxaline (red). R_7_ position showed the different substituent in each hybrid

Quinoxaline is an *N*-heterocyclic molecule composed of a benzene ring and a pyrazine ring [11]. Quinoxaline 1,4-di-*N*-oxide derivatives generated by oxidation of both nitrogen of this heterocyclic system have displayed therapeutic activity against different parasites as *Trypanosoma* [12, 13], *Leishmania* [14, 15], *Entamoeba histolytica* [16] and malarial activity against erythrocytic forms of *Plasmodium falciparum* [17–19]. Recently, primaquine–quinoxaline 1,4-di-*N*-oxide hybrids were synthetized and tested their activity against different *Plasmodium* life cycle stages. Two hybrids (**6a** and **6b**) showed in vitro activity against exoerythrocytic phase (IC_50_ < 6 mM) and sporogonic phase in *Anopheles stephensi* with 100 mg/kg dose at mice [20] (Fig. 1). However, the mode of action of these primaquine–quinoxaline 1,4-di-*N*-oxide (QdNO) hybrids is not fully understood. This study reports the mode of action (MA) of two new hybrids of primaquine linked to quinoxaline 1,4-di-*N*-oxide (PQ–QdNO) exhibiting potent anti-malarial activity against different stages of *Plasmodium* life cycle.

## Methods

### Parasites, cell line

*Plasmodium yoelii* 17XNL cryopreserved sporozoites were obtained in the Sanaria^®^ Company. HepG2/CD81 cells were given by Dr. D. Mazier and Dr. Olivier Silvie from Centre d’Immunologie et des Maladies Infectieuses UPMC Paris, France. The cells were cultivated in 96 well culture plate coated with rat tail collagen I (Advanced BioMatrix) at 37 °C under 5% CO_2_ in DMEM supplemented with 10% fetal calf serum and antibiotics (Sigma-Aldrich) as described in Bonilla-Ramirez et al. [20].

### PQ–QdNO hybrids

The new PQ–QdNO hybrids were synthesized using a three-step procedure [20]. Briefly, primaquine (PQ) was obtained from the commercially available primaquine bisphosphate through extraction using an aqueous solution of sodium bicarbonate to afford the free base of the compound. Subsequent acetoacetylation of PQ using diketene in the presence of methanol under a nitrogen atmosphere at 0 °C provided the β-acetoacetamide derivative. Finally, condensation of the acetoacetamide derivative with the corresponding benzofuroxans BFX (a–e) in the presence of calcium chloride and ethanolamine as catalysts by variation of Beirut reaction give the final primaquine–quinoxaline 1,4-di-*N*-oxide hybrids **6a**–**e**.

### *Plasmodium yoelii* infection and PQ–QdNO hybrids treatment of HepG2-CD81

HepG2/CD81 cells (3 × 10^4^ per well in collagen-coated 96-well plates) were infected with *P. yoelii* 17XNL (7 × 10^3^ spz per well) and cultured for 40 h before analysis. The in vitro antiplasmodial activity against the liver stage of PQ–QdNO hybrids (**6a**–**6e**) was recently reported [20]. Two PQ–QdNO hybrids (**6a** and **6b**) were chosen to perform assays about the mode of action. These compounds were selected based on better in vitro antiplasmodial activity and selectivity index in the liver stage. Primaquine was used as a reference drug in all experiments. Hybrids were diluted in DMEM and three specific concentrations of each hybrid (one-, two- and four-fold of its corresponding IC_50_) for tissue schizontocidal activity against *P. yoelii* in HepG2-CD81 cells were used for all the experiments. The treatment cell was simultaneous to infection. The culture medium was changed 3 h and every 24 h post-infection, and fresh compounds were added at the same concentration to maintain exposition. The cultures were allowed to grow at 37 °C in 5% CO_2_. After the time of developing for each parasite, the cells were analyzed according to the specific evaluation protocol. All experiments were performed in triplicate.

### Evaluation of the production of reactive oxygen species (ROS) in infected cultures HepG2-CD81 treated with hybrids **6a** and **6b**

The analysis of intracellular ROS production was carried out according to Bonilla-Porras et al. [21]. Briefly, 5 × 10^4^ cells were incubated with dichlorofluorescein diacetate (DCF-DA) at 10 μM final concentration for 25 min at 37 °C in the dark. Subsequently, the analysis of 1 × 10^4^ cells was performed by flow cytometry (Accuri C6 CSampler). All the experiments were performed in triplicate. The acquisition analysis was performed using the BD CSampler™ software. A non-parametric variance analysis (Kruskal–Wallis) and a Mann Whitney U for comparison between groups were performed. A confidence interval of 95% and a value a of 0.05 were considered for statistical significance.

### Determination of total glutathione content in infected cultures HepG2-CD81 treated with hybrids **6a** and **6b**

The HepG2-CD81 cells were cultured and treated as described. The concentration of total glutathione (tGSH, GSH + GSSG) in HepG2-CD81 cells parasitized and not parasitized with *P. yoelii* 17XNL and treated with PQ–QdNO hybrids **6a** and **6b** were evaluated. The analysis was performed as derivatives of mBBr (Thiolyte^®^, Calbiochem) by reverse phase HPLC. The method was carried out according to Zuluaga et al. [22]. 20 μL of sample were placed in 1.5 mL Eppendorf tubes placed on ice and protected from light, 10 μL of NaBH_4_ in a solution of 0.066 M NaOH and 33% DMSO (v/v), 6 μL of EDTA 2 mM plus 65 mM dithiothreitol solution, 6 μL of octanol and 14 μL of 1.8 M HCl was added. After 3 min, 70 μL of 1 M ethylmorpholine buffer (pH 8.5), 134 μL of deionized water and 14 μL of 5 mM Thiolyte were added. The derivatization was carried out at 70 °C for 10 min in the dark, and then 26 μL of 100% acetic acid was added. After 20 min on ice and in the dark, the sample was extracted with 200 μL of dichloromethane and centrifuged at 10,000 rpm at room temperature for 2 min. The supernatant (the water-soluble phase) was taken and filtered through a 0.45 μm nylon membrane. The filtered samples were stored at − 20 °C, protected from light until their injection into the HPLC. 20 μL from the water-soluble phase were injected in a reverse phase HPLC LiChroCART^®^ 100 RP-18 (5.0 μm). The column was eluted at a flow of 0.5 mL/min by the following gradient: solvent A (0.25% acetic acid) and solvent B (100% acetonitrile): 0 min, 100% solvent A; 5 min, 90% solvent A; 20 min, 85% solvent A, 25 min, 0% solvent A. The effluent was monitored by a fluorescence spectrophotometer (excitation at 400 nm, emission 475 nm). Under these conditions, the glutathione-Thiolyte adduct had a retention time of 30.5 min. A glutathione calibration curve was prepared in the samples and in the regression analysis (linear model). The sensitivity obtained was 3.2 pmol, which is in the range of quantity detected when working with compounds derivatized with monobromobimane. A non-parametric variance analysis (Kruskal–Wallis) and a Mann Whitney U for comparison between groups were performed. A confidence interval of 95% and a value a of 0.05 were considered for statistical significance.

### Analysis of the mitochondrial membrane potential in infected cultures HepG2-CD81 treated with hybrids **6a** and **6b**

The HepG2-CD81 cells were cultured and treated as described above. The mitochondrial membrane potential analysis was carried out at 2, 4, 24 and 48 h according to Bonilla-Porras et al. [21] with slight modifications. Briefly, 5 × 10^4^ cells/mL were incubated with the lipophilic cation 3,3′-dihexyloxacarbocyanine iodide (DiOC_6_(3), 40 nM, final concentration) for 15 min at 37 °C in the dark. Subsequently, the analysis of 1 × 10^4^ cells was carried out by flow cytometry (Accuri C6 CSampler). All the experiments were performed in triplicate. The acquisition analysis was performed using the BD CSampler™ software. A non-parametric variance analysis (Kruskal–Wallis) and a Mann Whitney U for comparison between groups were performed. A confidence interval of 95% and a value a of 0.05 were considered for statistical significance.

### Determination of the cell cycle and DNA fragmentation in infected cultures HepG2-CD81 treated with hybrids **6a** and **6b**

DNA fragmentation and the cell cycle were evaluated in HepG2-CD81 cells, which were cultured and treated as described. The method was performed as described in Bonilla-Porras et al. [23]. Briefly, after 40 h post infection and treatment, cells (5 × 10^4^) were washed twice with phosphate buffer solution (PBS, pH 7.2) and stored in 95% ethanol at − 20 °C. Before carrying out the reading, the cells were washed and incubated in 400 μL of the solution containing propidium iodide (PI, 50 μg/mL), RNAse A (100 μg/mL), EDTA (50 mM) and triton X-100 (0.2%) for 60 min at 37 °C. The cell suspension was analyzed for PI fluorescence using an Accuri C6 CSampler flow cytometer. Cells in the sub-G0/G1 phase were used as a marker of apoptosis. The cell cycle and DNA fragmentation were evaluated in two independent experiments. The quantitative data of each of the phases of the cell cycle and the figures of the sub-G0/G1 population were obtained using FlowJo 7.6.2 Data Analysis Software.

### Evaluation of cellular death markers by western blot in infected cultures HepG2-CD81 treated with hybrids **6a** and **6b**

To evaluate levels of protein expression related to oxidative stress (e.g. DJ-1oxy and GPx1), cell death (e.g. p53 and c-Jun) and the suggested mechanism of QdNO (e.g. HIF1a) were selected and their presence was evaluated in HepG2-CD81 cells not infected or infected with *P. yoelii* and treated with hybrids **6a** and **6b** or PQ as control. HepG2-CD81 cells infected or not infected with *P. yoelii* 17XNL and treated with PQ–QdNO hybrids **6a** and **6b** were cultured for 40 h at 37 °C and 5% CO_2_. After the incubation, cells were lysed in 50 mM Tris-HCl, pH 8.0, with 150 mM sodium chloride, 1.0% Igepal CA-630 (NP-40), 0.5% sodium deoxycholate and 0.1% sodium dodecyl sulfate and protease inhibitor cocktail (Sigma-Aldrich). The lysates of the samples were quantified using the bicinchoninic acid test (Thermo Scientific) and 40 mg of protein were loaded and separated using 12% electrophoresis gels, which were subsequently transferred to nitrocellulose membranes (Hybond-ECL, Amersham Biosciences) at 300 mA for 2 h using an electrophoretic transfer system (BIO-RAD). The membranes were incubated overnight at 4 °C with rabbit polyclonal anti-P53 (Santa Cruz Biotechnology, Inc.), rabbit polyclonal anti-c-Jun (Santa Cruz Biotechnology, Inc.), goat polyclonal anti-AIF (Santa Cruz Biotechnology, Inc.), rabbit anti-Cleaved polyclonal caspase-3 (Santa Cruz Biotechnology, Inc.) and mouse monoclonal anti-actin Clone C4 (Merck Millipore). The IRDye 800CW anti-rabbit and IRDye 680CW IRDye antibodies (LI-COR Biosciences, 1:5000) were used as secondary antibodies. The analysis was carried out using the Odyssey infrared imaging system (LI-COR Biosciences, Lincoln, NE, USA). A non-parametric variance analysis (Kruskal–Wallis) and a Mann Whitney U for comparison between groups were performed. A confidence interval of 95% and a value a of 0.05 were considered for statistical significance.

## Results

### Production of ROS in cells HepG2-CD81 infected with *Plasmodium yoelii* 17NXL and treated with PQ–QdNO hybrids

**Fig. 2:**
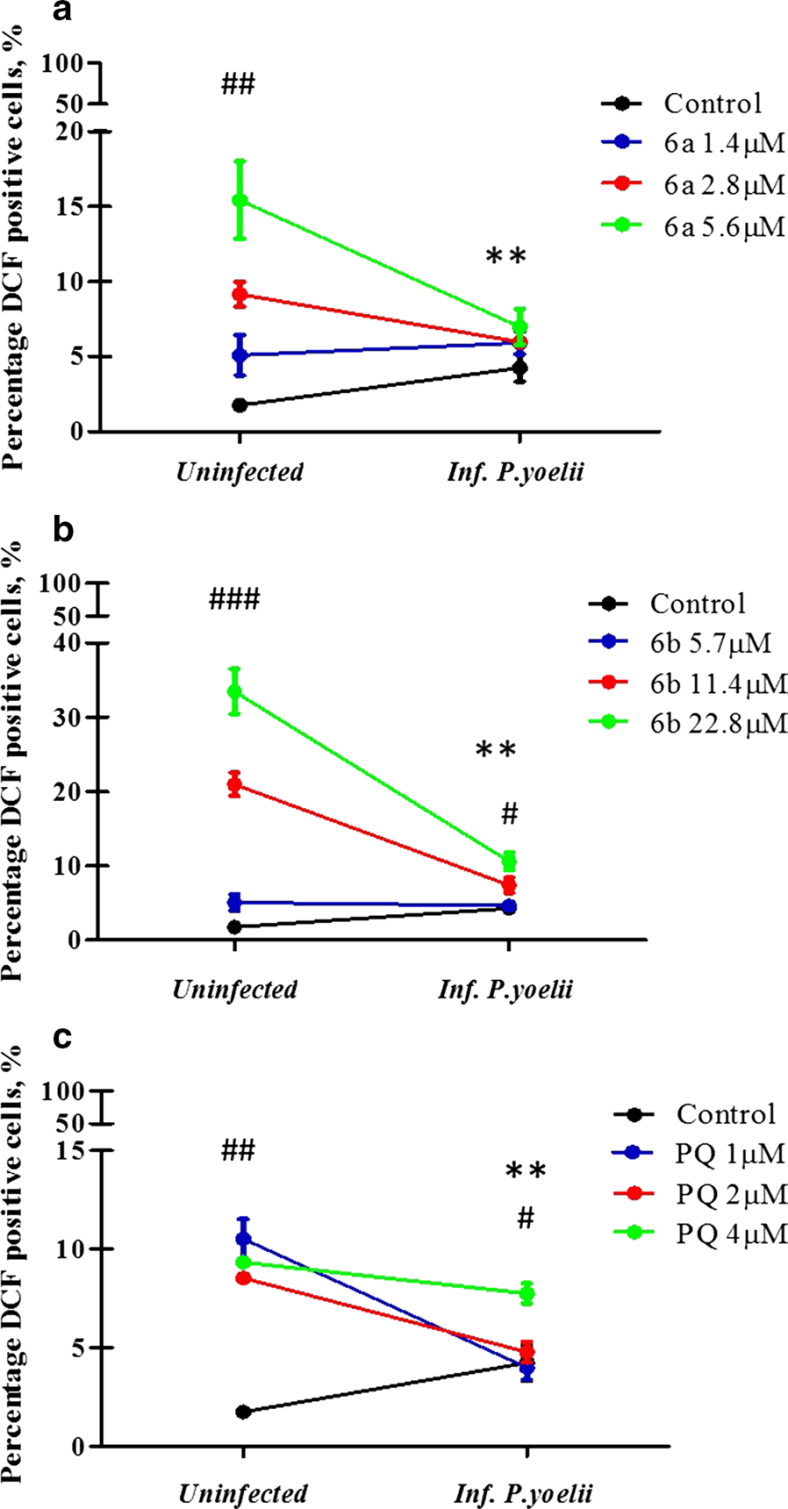
**6a** and **6b** PQ–QdNO hybrids induce production of ROS in HepG2-CD81 alone or infected with *P. yoelii* 17XNL. Line graphs (**a**–**c**) show the percentage of DCF positive HepG2-CD81 cells infected with *P. yoelii* 17XNL or uninfected and treated with hybrids **6a** (**a**), **6b** (**b**) and primaquine (**c**). The data are expressed as the average of three independent experiments. **p < 0.01 shows a statistically significant comparison between infected and uninfected groups. ^#^p < 0.05, ^##^p < 0.01, ^###^p < 0.001 show significant intra-group differences

The production of oxidative stress measured by the DCF-DA test showed that the treatment of the cells uninfected with hybrids **6a** (Fig. 2a) and **6b** (Fig. 2b) induced production of H_2_O_2_ in a dependent concentration manner. The process of infection of HepG2 cells by *P. yoelii* induced a slight decrease in ROS levels (Fig. 2). The treatment with the **6b** hybrid induced a concentration-dependent increase in both infected cells (p < 0.05) or uninfected (p < 0.01) (Fig. 2b), whereas infected with the hybrid **6a** showed slight increase in ROS production (Fig. 2a). However, no significant difference was found. The PQ induced oxidative stress independently of the concentration in uninfected cells and only at high concentration in infected cells (p < 0.01, Fig. 2b).

### Total glutathione content in cells HepG2-CD81 infected with *P. yoelii* 17NXL and treated with PQ–QdNO hybrids

**Fig. 3:**
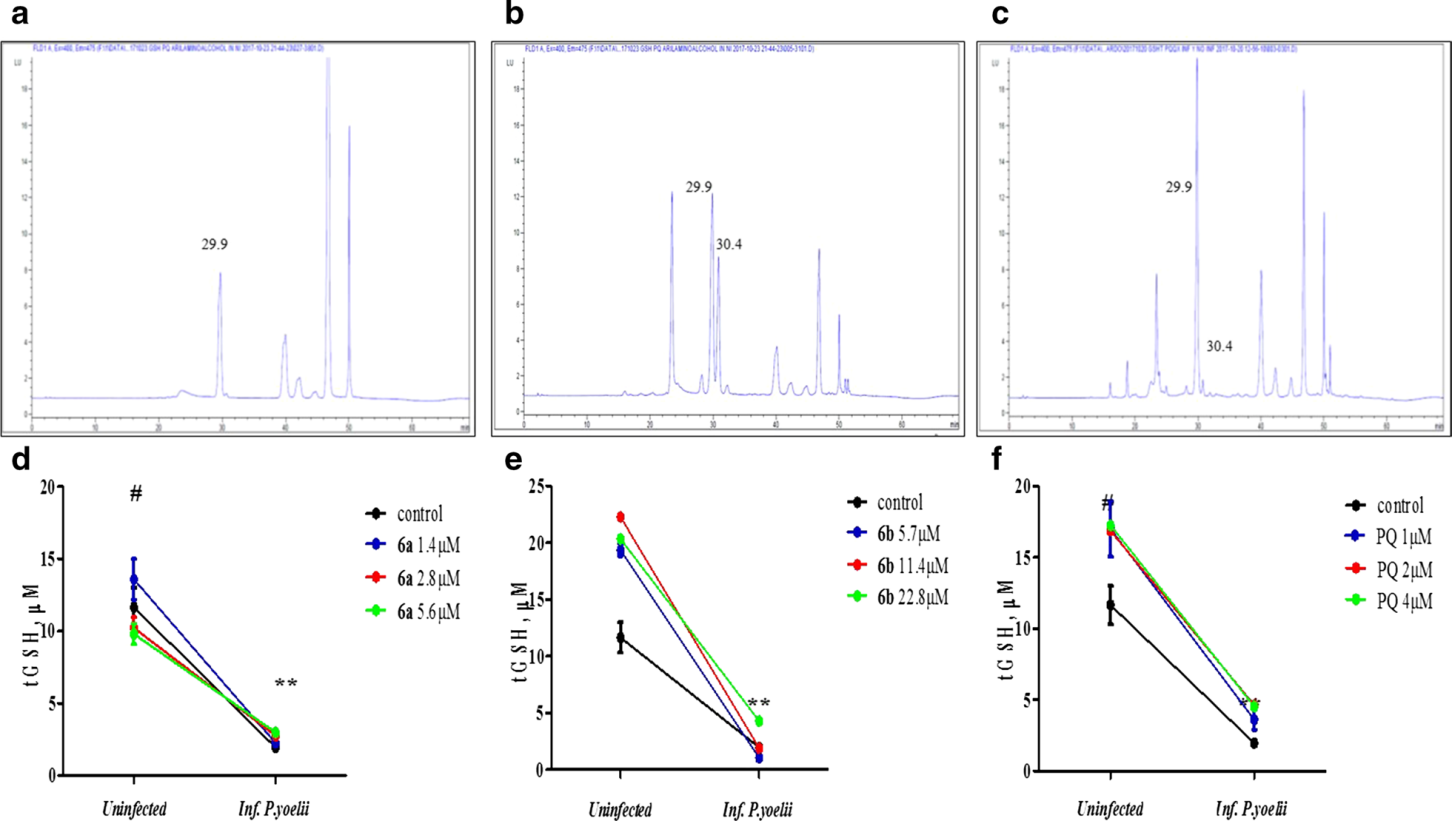
Determination of total Glutathione levels in HepG2-CD81 cells infected with *P. yoelii* 17XNL or uninfected and treated with hybrid PQ–QdNO compounds. Representative chromatograms and retention times (**a**–**c**) of Thiolyte and Thiolyte-GSH adducts. **a** Blank sample (cell-free), shows retention time (tr) of Thiolyte monobromobimane in 29.9 min of running. **b** Chromatogram of HepG2-CD81 uninfected control (Adduct Thiolyte-GSH tr: 30.4). **c** Chromatogram of HepG2-CD81 control infected with *P. yoelii* 17XNL (Adduct Thiolyte-GSH tr: 30.4). Line graphs (**d**–**f**) show the change in GSHt concentration in HepG2-CD81 cells infected with *P. yoelii* 17XNL or uninfected treated with PQ (**d**), and hybrids **6a** (**e**) and **6b** (**f**). The data are expressed as the average of three independent experiments. **p < 0.01 shows a statistically significant comparison between infected and uninfected groups. ^#^p < 0.05 shows significant differences between control and treated in the uninfected group

The content tGSH in HepG2-CD81 cells infected with *P. yoelii* 17XNL or uninfected and treated with hybrid compounds **6a** and **6b** was determined by measuring the fluorescent adduct Thiolyte-GSH by HPLC. The fluorescent signal to Thyolite was at 29.9 min of running (Fig. 3a) and the adduct Thiolyte-GSH showed a retention time at 30.4 min (Fig. 3b, c). Interestingly, infection of HepG2-CD81 with *P. yoelii* 17XNL reduced tGSH levels (p < 0.01) (Fig. 3d–f). The treatment of the cells without infection with the **6b** hybrid (Fig. 3f) and the primaquine (used as a control, Fig. 3d) increased the levels of tGSH compared to the control cells (p < 0.05) in the three concentrations evaluated. However, this increase was independent of concentration. On the other hand, treatment with the hybrid **6a** did not show any differences in tGSH levels in uninfected cells (Fig. 3e), whereas, in infected cells, this compound induced a slight increase in tGSH levels in a dependent manner of the concentration. This was evidenced in concentration 5.6 mM corresponding to fourfold higher than the IC_50_ for this hybrid. Interestingly, the hybrid **6b** showed a decrease in tGSH in infected cells at the concentrations corresponding to the IC_50_ (5.7 mM) and twice the IC_50_ (11.4 mM), while in uninfected cells this hybrid **6b** induced a contrary effect increasing the tGSH level in a manner depending on concentration (Fig. 3f). On the other hand, primaquine in infected cells increased tGSH levels in a concentration-dependent manner (Fig. 3d).

### Potential of mitochondrial membrane (DYm) in cells HepG2-CD81 infected with *P. yoelii* 17NXL and treated with PQ–QdNO hybrids

In the evaluation of mitochondrial membrane potential in uninfected HepG2-CD81 cells after treatment with **6a**, **6b** hybrids and PQ, mitochondrial depolarization in 2, 4 and 24 h after treatment was not observed. All treatments independently of concentration and exposure time showed 100% of positive cells to DiOC_6_(3).

**Fig. 4:**
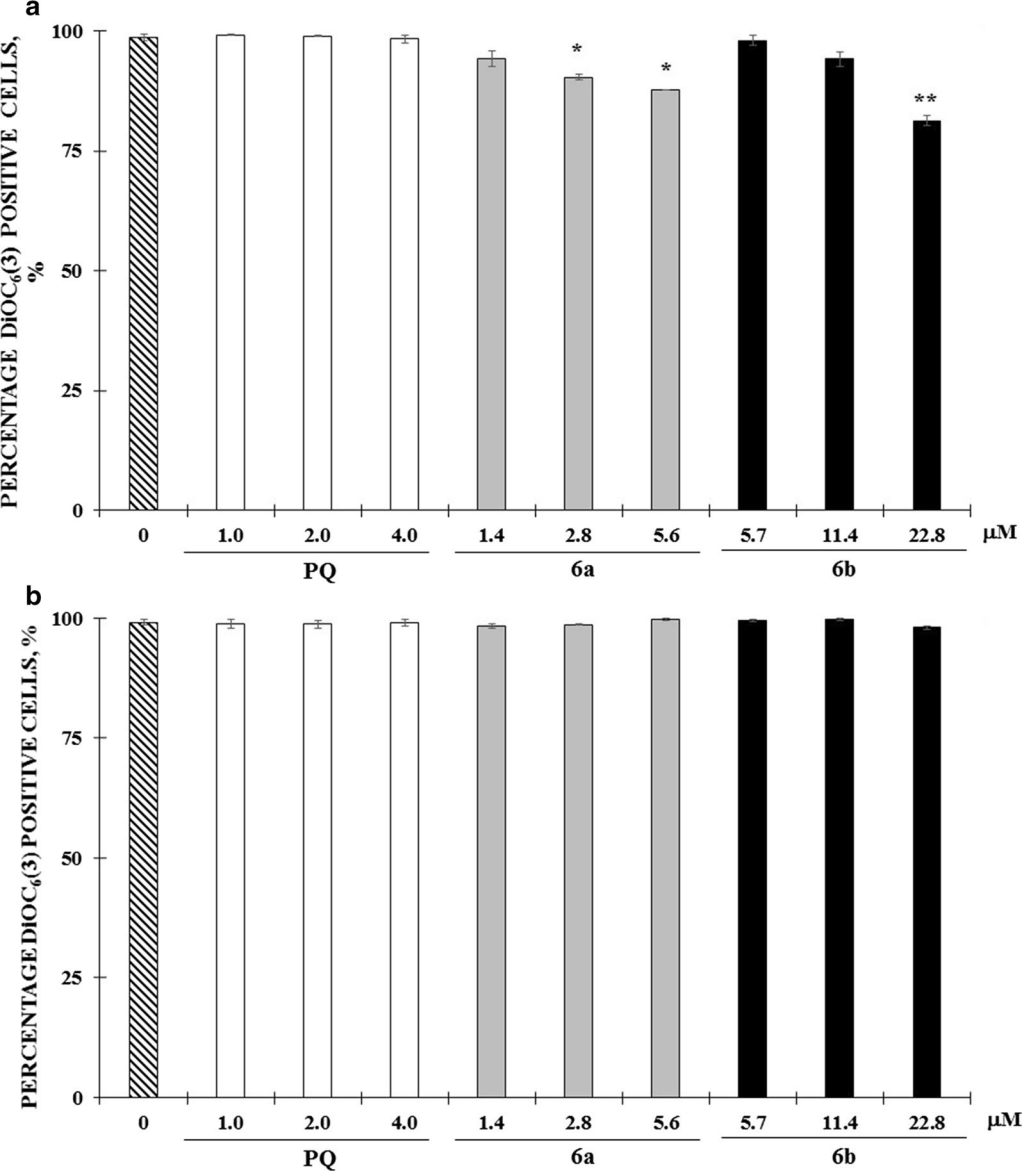
Mitochondrial membrane potential (DYm) of HepG2-CD81 was not disturbed by infection *P. yoelii* 17XNL or treatment with 6a and 6b PQ–QdNO hybrids. Bar graphs (**a**, **b**) show the percentage of DiOC6 (3) positive HepG2-CD81 cells for in the uninfected groups (**a**) or infected with *P. yoelii* 17XNL (**b**) and treated with **6a**, **6b** hybrids, and primaquine. The data are expressed as the mean of three independent experiments ± SD. *p < 0.05 and **p < 0.01 shows statistically significant differences compared to the control group

Only, a concentration-dependent reduction was observed at 48 h of treatment with **6a** (20%) and **6b** hybrids (25%) (Fig. 4a). The mitochondrial membrane potential of HepG2-CD81 cells was not affected in the process of infection with *P. yoelii* or by treatment with the hybrids evaluated (Fig. 4b).

### Cell cycle and nuclear fragmentation in cells HepG2-CD81 after infection with *P. yoelii* 17XNL and treated with PQ–QdNO hybrid

**Fig. 5:**
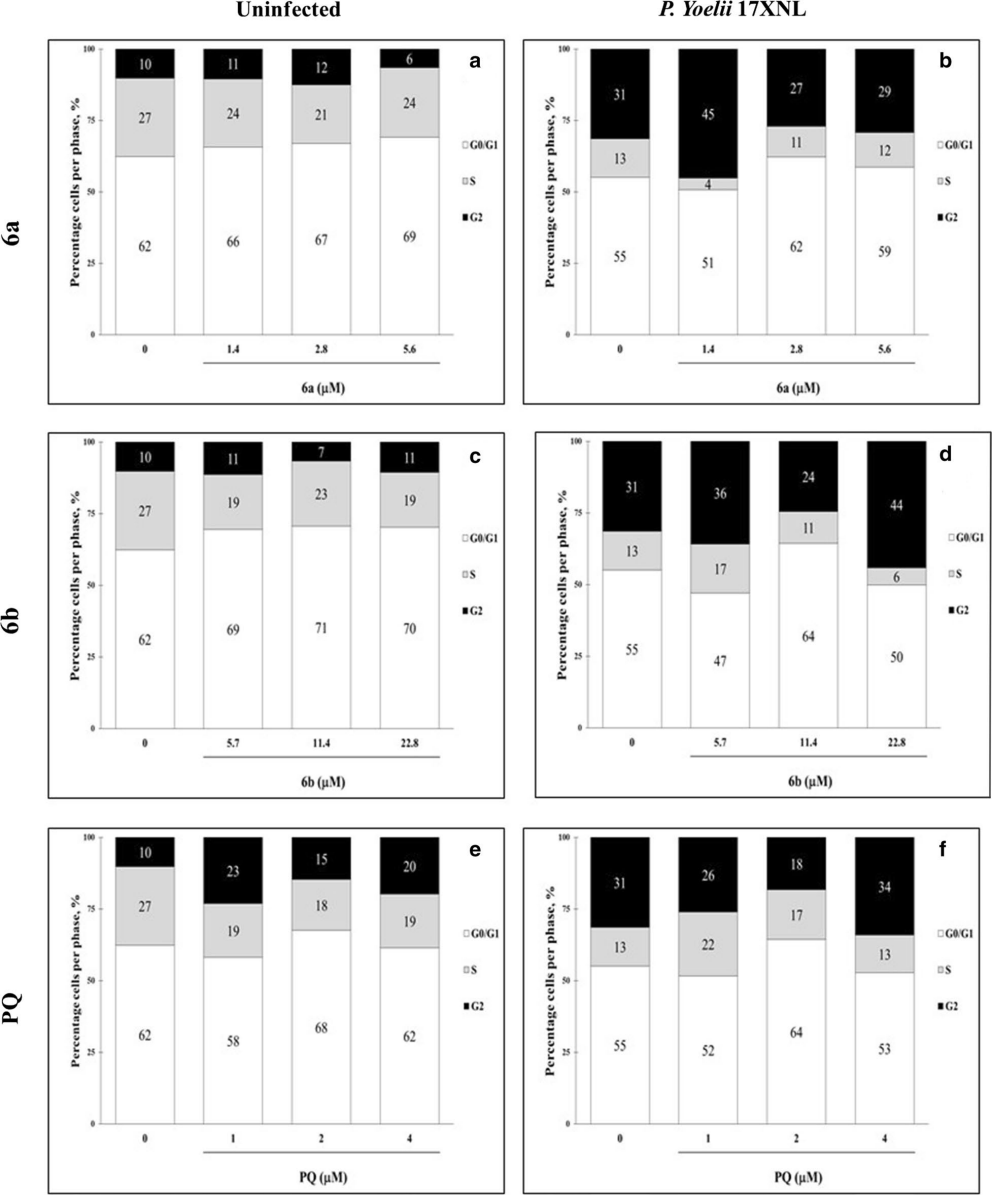
Cell cycle analysis of HepG2-CD81 cells infected or not with *P. yoelii* 17XNL treated with PQ–QdNO hybrid. Stacked bar graphs show the percentages of each phase of the cell cycle (G0/G1: white; S: gray; G2: black) in uninfected HepG2-CD81 cells (**a**, **c**, **e**), and infected with *P. yoelii* 17XNL, after 48 h of infection and treatment with hybrid compounds **6a** (**a**, **b**), **6b** (**c**, **d**) and PQ (**e**, **f**). The data are expressed as the average of three independent experiments. The percentage of each phase was calculated with Jean-Fox’s cell cycle analysis model

**Fig. 6:**
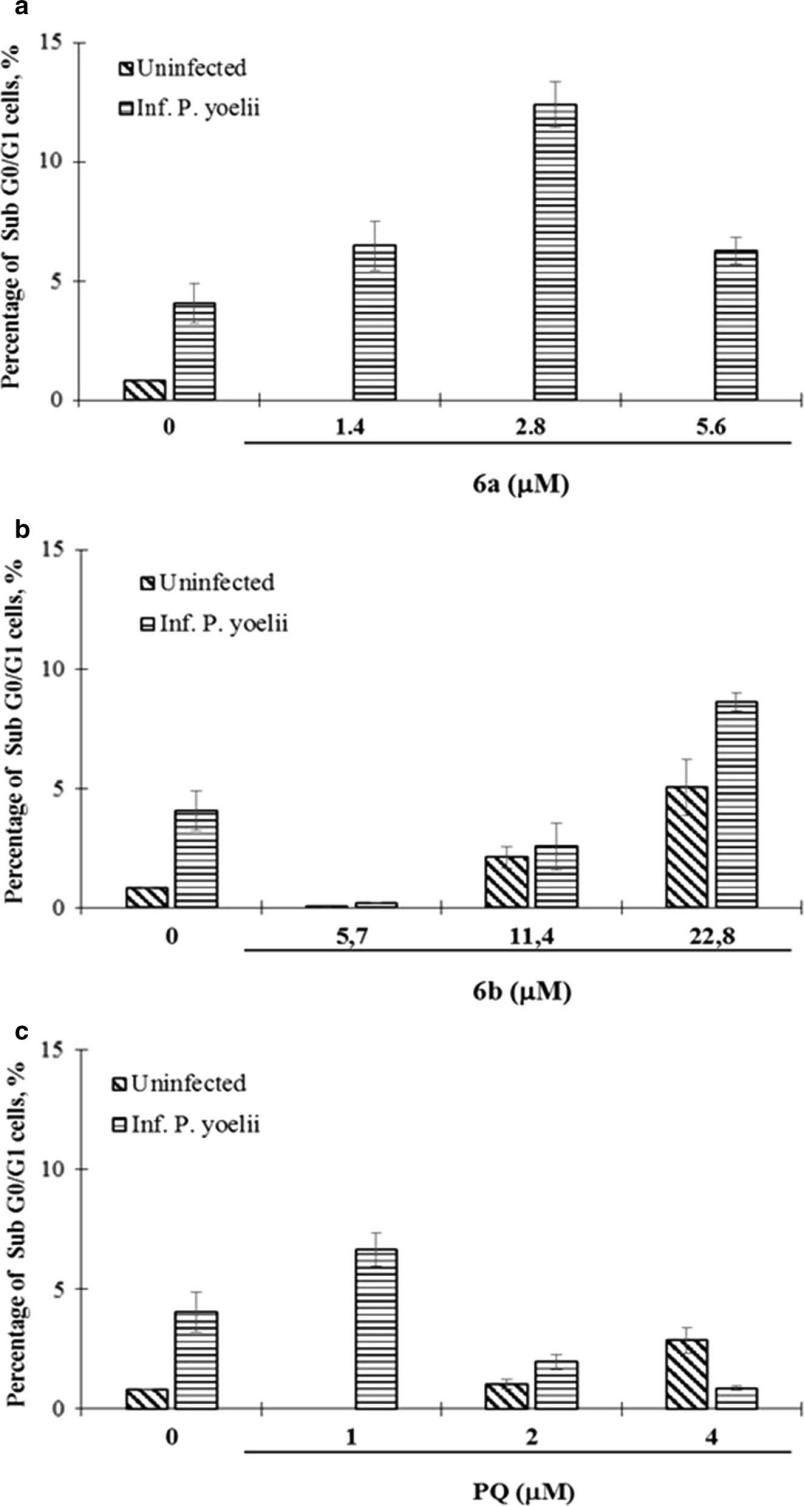
Analysis of nuclear fragmentation in HepG2-CD81 cells treated with hybrid PQ–QdNO compounds and infected with *P. yoelii* 17XNL. Bar graphs show the percentages of nuclear fragmentation (**a**–**c**), after 48 h of infection with *P. yoelii* 17XNL and treatment with **6a** (**a**), **6b** (**b**) hybrid and PQ (**c**). The data are expressed as the mean of three independent experiments ± SD. The fragmentation percentage was calculated with Jean-Fox’s cell cycle analysis model

Hybrids **6a** and **6b** induced cell cycle arrest in uninfected HepG2-CD81 cells, evidenced by the increase in the G0/G1 phase and reduction of the synthesis phase, without modification of the G2 phase of the cell cycle (Fig. 5a, c). Treatment with PQ in these naive cells did not show evidence of cell cycle arrest. On the contrary, PQ increased the G2 phase of the cells exposed to this drug (Fig. 5e). The infection of HepG2-CD81 cells with *P. yoelii* 17XNL surprisingly favors cell cycle progression (Fig. 5b). However, it induces nuclear fragmentation (Fig. 6). Interestingly, the treatment with all the compounds evaluated does not affect this progression of the cell cycle to G2 phase induced by the infection regardless of the concentration of the compound evaluated (Fig. 5b, d and f). On the other hand, uninfected cells treated with the hybrid **6a** did not show nuclear fragmentation, but increasing of fragmentation was observed when the cell was infected with *P. yoelii* (Fig. 6a). Likewise, the hybrid **6b** induced nuclear fragmentation in a concentration-dependent manner, but independent of *P. yoelii* infection (Fig. 6b). On the other hand, PQ presented a dual behavior inducing concentration-dependent nuclear fragmentation in uninfected cells and inversely proportional to the concentration in cells infected by *P. yoelii* (Fig. 6c).

### Preliminary evaluation of cellular death signaling in HepG2-CD81 infected with *P. yoelii* induced by **6a** and **6b** PQ–QdNO hybrids

**Fig. 7:**
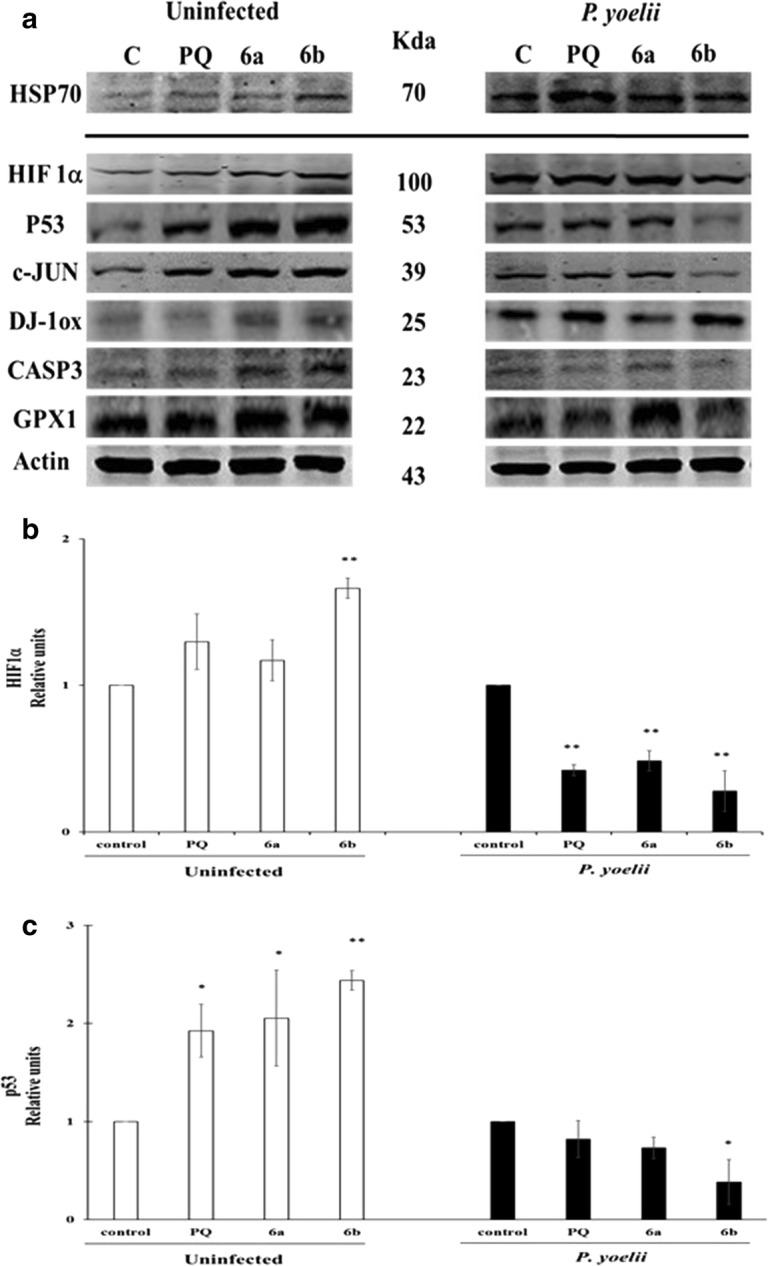

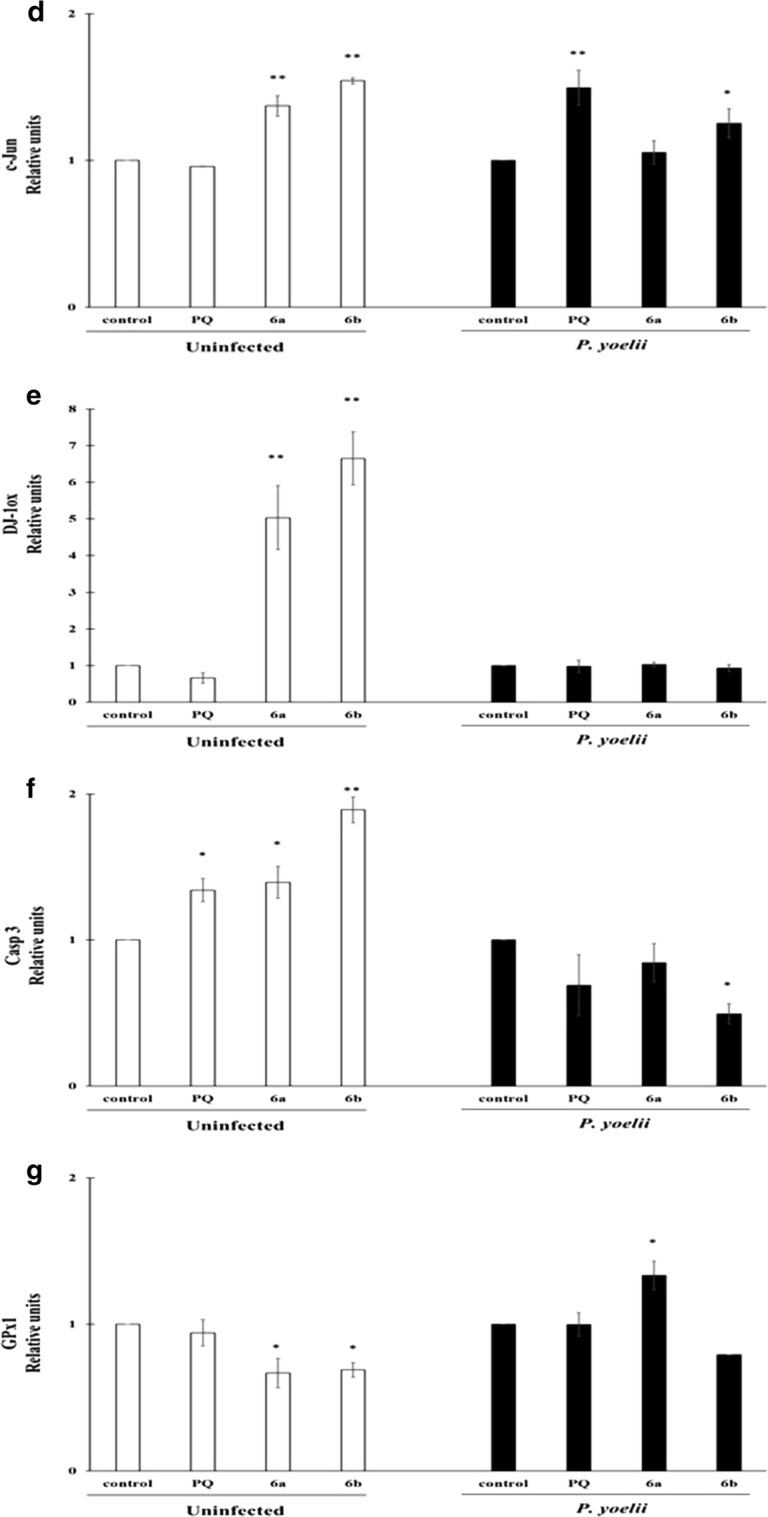
Molecules related to oxidative stress and cell death involved in the tissue schizontocidal activity of PQ–QdNO hybrids in HepG2-CD81 cells infected with *P. yoelii* 17XNL. **a** Representative image of western blot shows the expression of P53, c-JUN, oxidized DJ-1 (DJ-1ox), HIF1a, CASP3, glutathione peroxidase 1 (GPX-1) in uninfected HepG2CD81 cells or infected with *P. yoelii* 17XNL and treated or not with PQ–QdNO hybrids. The mean intensity value of the bands shown in (**a**) was measured by an infrared imaging system (Odyssey, LI-COR), and the intensity was normalized to that of Actin (**b**–**g**). The data are expressed as the mean ± standard deviation of three independent experiments. *p < 0.05 and **p < 0.01 shows statistically significant differences compared to the control group

To contribute to the elucidation of the cell death signaling cascade induced by the PQ–QdNO hybrids **6a** and **6b** as tissue schizontocides, several molecules related to oxidative stress, cell death and the suggested mechanism of QdNO were selected. The Hsp70 protein was evaluated to verify the infection. Interestingly, there was a weak cross-reactivity with a 70 kDa protein of the HepG2-CD81 cells. The western blot analysis revealed that in uninfected cells the hybrids **6a** and **6b** increase the levels of all the proteins evaluated (Fig. 7a–f) except for GPx1, which decreases in the cells treated with these compounds.

## Discussion

The QdNO have shown multiple biological activities [24], such as antibacterial [25, 26], antimycobacterial [27–29] and anticancer [30–32]. For this reason, QdNO derivatives present potential perspectives in several fields of human medicine, such as parasitology. The preliminary analysis of the mode of action of hybrids **6a** and **6b** in the exoerythrocytic phase showed that the infection of HepG2-CD81 cells with *P. yoelii* 17XNL induces production of ROS. This result is in agreement to the study reported by Delhaye et al. who explained that the increase in oxidative stress production could be due to an increase in the energy requirement in infected individuals [33]. Additionally, this increased ROS production could be explained as the result of the activation of the immunity, functions that can be energetically expensive [33], or to the deviation of energy by the parasite for its own development [34]. This increased production of stress may be closely related to the observed decrease in the total GSH concentration in the infected cells, where there was an expenditure of glutathione, without renewal of the same, which may suggest that glutathione anabolism can be inhibited within the host cell during the infection process, or that the rate of use of glutathione is higher than that of glutathione production. In contrast, in intact cells, the treatment with hybrids **6a** and **6b** and PQ showed an increase in the concentration of glutathione suggesting a compensation response to H_2_O_2_ [35]. Interestingly, the hybrid **6a** treated with doses higher than its IC_50_ induced a decrease in tGSH. This fact suggests a difference in the modes of action of the two hybrids that must be the subject of future research. Moreover, in cells infected with *P. yoelii* 17NXL, the hybrid **6a** and the PQ increased the levels of tGSH, while the hybrid **6b** did not, despite the difference in concentration to reach the IC_50_, and then in the case of the **6b** hybrid is 4 times higher compared to **6a** hybrid. This result suggests that this compound induces less oxidative stress and, in consequence, this may be the reason why it presents a lower anti-malarial activity against the exoerythrocytic forms of *P. yoelii* spp. In turn, this finding suggests that, although the hybrid **6b** induces oxidative stress, it may be acting by other signaling pathways to induce the death of exoerythrocytic parasitic forms; these signaling pathways may be inhibited by *P. yoelii* 17XNL since **6b** showed higher activity when it was tested against *Plasmodium berghei* and *P. falciparum*.

On the other hand, the results obtained suggest that parasite death induced by the hybrids **6a** and **6b** and even PQ are independent of mitochondrial signaling. A slight mitochondrial depolarization induced by hybrids was observed in uninfected cells, but once the process of infection by *P. yoelii* 17XNL occurs, there is a preservation of the mitochondrial membrane potential. This fact is consistent with the non-activation of the transcription factor p53 and caspase effector of apoptotic cell death, caspase 3 in infected cells and treated with the hybrids. Moreover, the hybrid **6b** showed a negative regulation of p53, which is consistent with a higher probability of infection according to Kaushansky et al. [36], since p53 was suppressed during the infection process. This result supports the notion that **6b** induces its tissue schizontocidal activity in *P. yoelii* spp., by other mechanisms, since it is unable to induce the activation of p53 in the host cell.

These phenomena of cell survival can be due to mechanisms of adaptation of the niche of the host cell to their specific needs [36]. This approach is in agreement with the data observed at the cell cycle level, where a progression of the cycle was observed when comparing infected and uninfected cells. This phenomenon is not an unusual event. It has been widely reported in pathogenic organisms of the Apicomplexa group as *Toxoplasma gondi* [37], *Theileria annulata* and *Theileria parva* [38]. Moreover, this manipulation of the cell cycle of the host cell can be fundamental in the exoerythrocytic phase of *Plasmodium* spp., since it must guarantee the replicative success of a sporozoite to thousands of merozoites [39]. In addition, the progression of the cell cycle in the host cell would favor the increase in the number of cellular organelles that lead to greater availability of resources for the parasite during its development [35]. This is consistent with the observations of infection in polyploid cells reported by Austin et al. [40].

On another side, in the analysis of nuclear fragmentation high levels of cells in the subG0/G1 state were found. This increase was the concentration-dependent manner of hybrid evaluated. This result along with the activation of the transcription factor c-Jun favors the hypothesis of a mechanism of death derived from oxidative stress independent of mitochondria. Even more, taking into account: (i) the oxidation found of oxidative stress sensor protein DJ-1, a phenomena recognized after an exposure to molecule that induces oxidative stress [40, 41]; (ii) the alteration of glutathione levels; and (iii) the classic activation mechanism of c-Jun, mediated by ASK-1, a kinase activated by oxidative stress through JNK [42–44]. The down-regulation of factor 1a induced by hypoxia (HIF1a) has been reported as part of the mechanism of action of QdNO [11]. This transcription factor was negatively regulated in the infected cells, similarly to anticancer activity. This downregulation may mediate the control exerted by the parasite on the infected host cell. Interestingly, HIF1a was activated in uninfected cells.

## Conclusions

The results obtained in vitro in this work showed that the PQ–QdNO hybrids **6a** and **6b** exert their antiplasmodial activity in the exoerythrocytic phase by generating a cellular microenvironment with high levels of oxidative stress, which promotes the increase of total glutathione levels and oxidation stress sensor protein DJ-1. These mechanisms are understood as a compensatory response to an oxidizing cellular condition. In addition, this data confirms the role of HIF1a on QdNO action independent of biological activity. However, PQ–QdNO hybrids did not activate the transcription factor p53 related to cell death. As a consequence, it is necessary to continue deepening in the modes of action of these molecules with promising potential as anti-malarial agents and, thus continue in the process of design and development of more effective molecules that allow contributing in the process of control and elimination of malaria.

## Data Availability

The data and results obtained in the present study are available from the corresponding author upon request.

## Abbreviations

ACT: artemisinin-based combination therapy
ASK-1: kinase 1 activated by oxidative stress through JNK
DCF-DA: dichlorofluorescein diacetate
DiOC_6_(3): 3,3′-dihexyloxacarbocyanine iodide
EEF: exoerythrocytic forms
tGSH: total glutathione (GSH + GSSG)
HIF1a: factor 1a induced by hypoxia
IC_50_: inhibitory concentration 50%
PI: propidium iodide
PQ: primaquine
PQ–QdNO: primaquine linked to quinoxaline 1,4-di-*N*-oxide
QdNO: quinoxaline 1,4-di-*N*-oxide
ROS: reactive oxygen species
